# The biomimetic synthesis of balsaminone A and ellagic acid via oxidative dimerization

**DOI:** 10.3762/bjoc.16.169

**Published:** 2020-08-18

**Authors:** Sharna-kay Daley, Nadale Downer-Riley

**Affiliations:** 1 Department of Chemistry, The University of the West Indies, Mona, Jamaica

**Keywords:** balsaminone A, biomimetic synthesis, ellagic acid, oxidative dimerization

## Abstract

The application of oxidative dimerization for the biomimetic synthesis of balsaminone A and ellagic acid is described. Balsaminone A is synthesized via the oxidative dimerization of 1,2,4-trimethoxynaphthalene under anhydrous conditions using CAN, PIDA in BF_3_**·**OEt_2_ or PIFA in BF_3_**·**OEt_2_ in 7–8% yields over 3 steps. Ellagic acid is synthesized from its biosynthetic precursor gallic acid, in 83% yield over 2 steps.

## Introduction

**Figure 1:**
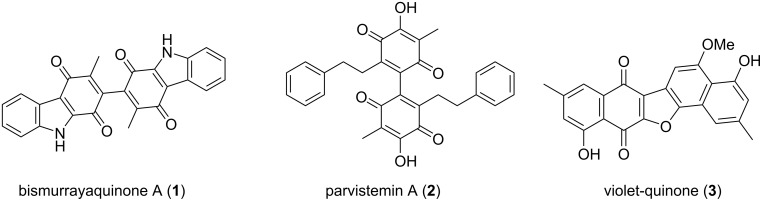
Selected natural products synthesized via oxidative dimerization.

Over the last century, the formation of an aryl to aryl bond has garnered considerable synthetic attention due to the applications of biaryls as pharmaceutical agents, as well as chiral auxiliaries in asymmetric synthesis [1–3]. Methods such as the Ullman coupling, Suzuki–Miyaura coupling and Stille coupling have dominated the field for the synthesis of biaryls [1,4]. Throughout the years, the exploration of oxidative dimerization reactions of electron-rich aromatic compounds, such as thiophenes, anilines and alkoxyarenes, in an attempt to establish high-yielding and selective oxidative coupling reactions, has afforded new and greener synthetic protocols for biaryls [5–7]. Several oxidants, such as the salts of Ag(I&II) [8], Ti(III&IV) [9], Mn(III) [10], Ce(IV) [11], Sn(IV) [12] and Fe(III) [13], as well as the hypervalent iodine reagents phenyliodine diacetate (PIDA) and phenyliodine bis(trifluoroacetate) (PIFA), have been utilized for oxidative dimerization reactions [14–15]. The use of these one-electron oxidants, as well as non-metallic reagents, plays an important role in accessing symmetrical and asymmetrical biaryls and polyaryls [1]. There are many important applications of oxidative dimerization reactions, one of which is the direct synthesis of natural product scaffolds [1,7,16–18]. Examples of this application include the biomimetic synthesis of the bioactive natural products bismurrayaquinone A (**1**) [16], parvistemin A (**2**) [17] and violet-quinone (**3**) [18] (Figure 1).

**Scheme 1:**
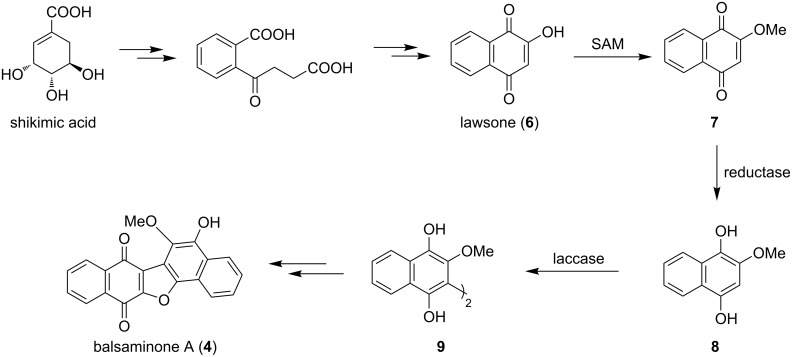
Proposed biosynthesis of balsaminone A (**4**) [19].

**Scheme 2:**
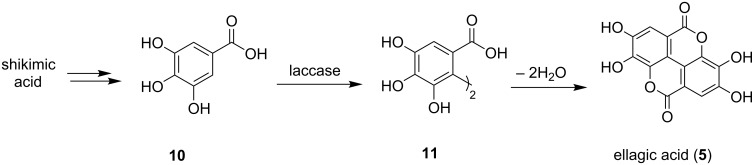
Proposed biosynthesis of ellagic acid (**5**) [20].

Similar to those natural products, the biomimetic synthesis of balsaminone A (**4**) and ellagic acid (**5**) can be attained using oxidative dimerization reactions, based on their proposed biosynthesis (Scheme 1 and Scheme 2) [19–20]. These pathways, which start with shikimic acid, feature the dimerization of monomeric phenolic precursors by a laccase enzyme, a single-electron enzyme complex within macro-organisms which facilitates oxidative dimerization through phenolic coupling [19]. In the case of balsaminone A (**4**), lawsone (**6**) is methylated to ether **7** which undergoes reduction to the dihydroxynaphthalene **8**. This is then dimerized by the copper-rich enzyme to quinone arenol **9**, which, following intramolecular cyclization, affords balsaminone A (**4**, Scheme 1). Similarly, the biosynthetic precursor of ellagic acid (**5**), gallic acid (**10**), undergoes dimerization to the dimeric polyphenolic acid **11**, followed by intramolecular cyclization to yield ellagic acid (**5**, Scheme 2) [20].

**Scheme 3:**
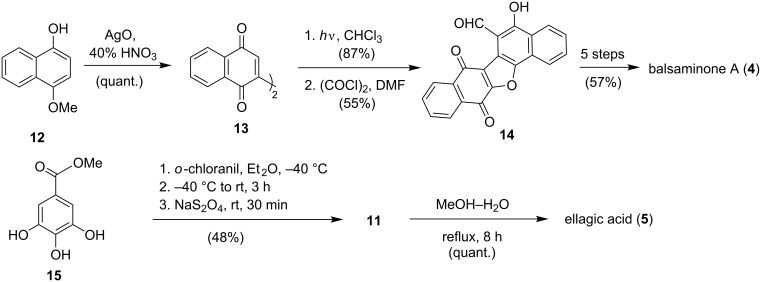
Previous syntheses of balsaminone A (**4**) [22] and ellagic acid (**5**) [23].

Because of the bioactivity of both natural products, there have been previous investigations of the synthesis of balsaminone A (**4**) [21–22] and ellagic acid (**5**) [23]. In our previous synthesis of balsaminone A (**4**) [22] (Scheme 3), silver oxide was used as the oxidative dimerizing agent for the formation of the binaphthoquinone intermediate **13**. This was followed by photolytic cyclisation and *ortho*-formylation to give carbaldehyde **14**. Conversion to balsaminone A (**4**) could then be achieved in 57% yield over 5 steps. The synthesis of ellagic acid (**5**) on the other hand featured an *o*-chloranil-mediated dimerization of methyl gallate (**15**), which yielded the natural product in 48% yield over 5 steps, as reported by Tsuboi et al. (Scheme 3) [23].

## Results and Discussion

### Biomimetic synthesis of balsaminone A

**Scheme 4:**
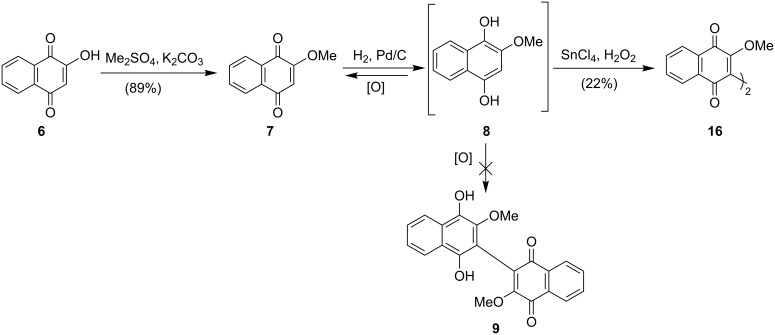
Attempted synthesis of the biomimetic precursor **9**. [O]: Act-C, K_3_[Fe(CN)_6_], or *p*-benzoquinone.

With the initial focus being the biomimetic synthesis of balsaminone A (**4**), well-established methods for the dimerization of phenolic compounds were explored [8–9]. As such, a methanolic solution of 2-methoxy-1,4-dihydroxynaphthalene (**8**), obtained from the reduction of lawsone (**6**), was subjected to the oxidants activated carbon (Act-C), potassium ferricyanide (K_3_[Fe(CN)_6_]), *p-*benzoquinone and stannic chloride (SnCl_4_). As shown in Scheme 4, with the exception of SnCl_4_, all oxidants resulted in the re-oxidation of the hydroxylated substrate to naphthoquinone **7**. SnCl_4_, however, while not successful for the synthesis of the desired biomimetic precursor **9**, afforded the binaphthylquinone **16** in 22% yield.

Given the initial unsuccessful attempt at the synthesis of the biomimetic precursor of balsaminone A (**4**), it was evident that alternative oxidative dimerization conditions needed to be explored. The oxidants cerium(IV) ammonium nitrate (CAN), ferric chloride hexahydrate (FeCl_3_·6H_2_O), vanadium pentoxide (V_2_O_5_), PIFA, and PIDA, in addition to SnCl_4_, were considered. Also investigated were 2-iodoxybenzoic acid (IBX) because of its implication in single-electron oxidation [24], and chromium trioxide (CrO_3_), which, based on its Cr(VI) oxidation state, should be able to facilitate single-electron transfer in the presence of electron-rich arenes.

The dimerization of 1,2,4-trimethoxynaphthalene (**17**) in the presence of the metal oxidants CAN, V_2_O_5_, and CrO_3_, afforded binaphthyl **16** in 29, 34, and 19% yields, respectively (Table 1). As anticipated, the reaction of naphthalene **17** with CAN under aqueous conditions resulted in preferential oxidative demethylation to give quinone **7**, as opposed to oxidative dimerization. The elimination of water from the reaction posed a few challenges since the solubility of CAN in organic solvents determines the likelihood of nitration or oxidative dimerization [1]. It was found that dry acetonitrile was more conducive to nitration, whereas methanol afforded the desired biaryl in 55% yield. In contrast to CAN, both V_2_O_5_ and CrO_3_ required aqueous conditions to prevent complexation of the reagent and the starting material. Of the Lewis acids used, stannic chloride proved to be the most effective oxidant for dimerization (Table 1). However, the hypervalent iodine reagents PIFA and PIDA gave better results overall, affording dimer **18** in 63% and 59% yields, respectively (Table 1).

**Table 1:** Reagents and products in the oxidative dimerization of 1,2,4-trimethoxynaphthalene (**17**).

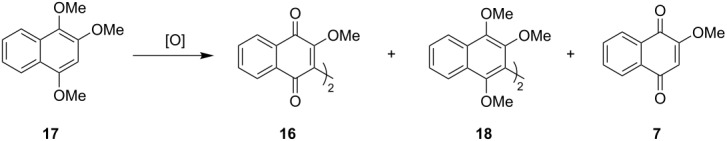			

	**16** (%)	**18** (%)	**7** (%)

CAN^a^	29^b^	55^c^	67^b^
V_2_O_5_^a,b^	34	–	–
CrO_3_^a,b^	19	–	62
FeCl_3_·SiO_2_^a,c^	15	43	–
FeCl_3_·6H_2_O^a,c^	–	8	–
SnCl_4_^d^	22	48^d^	–
PIDA, BF_3_·OEt_2_^e,f^	21	59	–
PIFA, BF_3_·OEt_2_^e,f^	12	63	–
IBX, BF_3_·OEt_2_^e,f^	13	–	45

^a^Reaction of naphthalene **17** (0.5 mmol) with oxidant (1.65 mmol) at rt. ^b^Addition of oxidant in H_2_O (1 mL, dropwise) to substrate in CH_3_CN or CH_3_OH (1 mL). ^c^Addition of oxidant (neat) in thirds, to substrate dissolved in CH_3_CN. ^d^Reaction was carried out in CH_2_Cl_2_ at 100 °C in a sealed tube. ^e^Reaction of naphthalene **17** (0.5 mmol) with oxidant (0.65 mmol). ^f^Reaction carried out under N_2_.

**Scheme 5:**
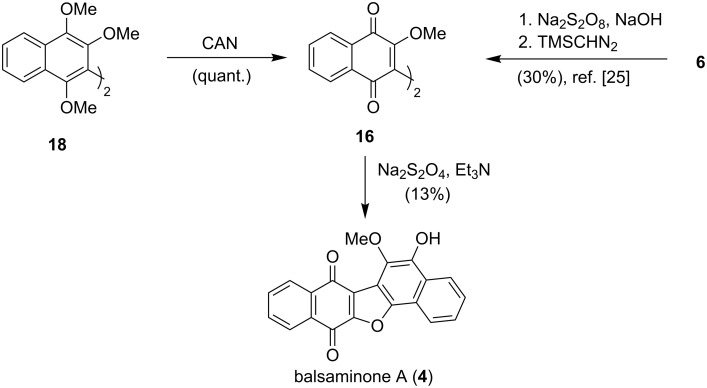
Biomimetic synthesis of balsaminone A (**4**).

Following the successful oxidative dimerization of 1,2,4-trimethoxynaphthalene **17** to biaryl **18**, the biaryl was converted quantitatively to binaphthyl **16** in the presence of an aqueous solution of CAN at 0 °C. Alternatively, the binaphthylquinone **16** was synthesized through the oxidation of lawsone (**6**), in the presence of sodium persulfate, followed by methylation [25]. The cyclization of binaphthyl **16** was then attempted taking into consideration the photolytic cyclization of binaphthyls to form pentacyclic furan derivatives [8,18,22]. However, the photolytic cyclization of binaphthyl **16** using a 100 W bulb was attempted without success, due to degradation of the starting material. This led to a different approach employing a reductive, base-mediated cyclization using alkaline aqueous sodium dithionite. Interestingly, the typical conditions for reduction using dithionite proved too harsh for the substrate, and like the photolysis of the binaphthyl **16**, degradation occurred. However, the use of triethylamine instead of aqueous sodium hydroxide resulted in the isolation of balsaminone A (**4**) in 13% yield, an overall 7–8% yield over 3 steps, completing the biomimetic synthesis of the natural product as shown in Scheme 5.

### Synthesis of ellagic acid

**Scheme 6:**
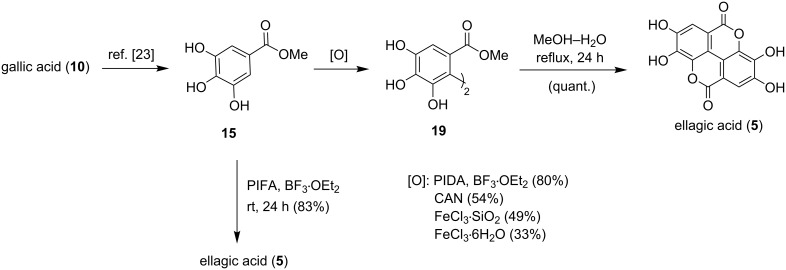
Concise and efficient biomimetic synthesis of ellagic acid (**5**).

With the synthesis of balsaminone A (**4**) accomplished, the direct biomimetic synthesis of ellagic acid (**5**) was targeted. While the synthesis of ellagic acid (**5**) has been achieved in 48% yield over four steps by Alam et al. [23], starting from methyl gallate (**15**), it was anticipated that a more concise and efficient synthesis could be attained. Methyl gallate (**15**), which may be obtained commercially or from the methylation of gallic acid (**10**) [23], was subjected to the oxidants CAN, PIDA, FeCl_3_·6H_2_O, and FeCl_3_·SiO_2_. Treatment of the polyphenol with PIDA (1.5 equiv) in boron trifluoride etherate (1.5 mL) at room temperature proved to be the most successful dimerization protocol, as the dimeric precursor **19** was formed in 80% yield after 16 hours. The biaryl **19** was converted quantitatively to the natural product when heated at reflux in aqueous methanol (1:1) for 24 hours. However, allowing the PIFA reaction mixture to stir at room temperature for 24 hours resulted in complete cyclization, affording the natural product **5** in 83% yield directly from methyl gallate (**15**, Scheme 6), an improvement for the synthesis of the bioactive natural product.

## Conclusion

The synthesis of balsaminone A (**4**) is reported in 7–8% yield over 3 steps from 1,2,4-trimethoxynaphthalene (**17**). Also, the one-step synthesis of ellagic acid (**5**) from methyl gallate (**15**) in 83% yield is described. These biosynthetically-driven syntheses represent the most efficient routes to these bioactive natural products to date.

## Supporting Information

File 1 Experimental part.
