# Supplementary material for: The biomimetic synthesis of balsaminone A and ellagic acid via oxidative dimerization
Source: Beilstein J Org Chem. 2020 Aug 18;16:2026–31. doi: 10.3762/bjoc.16.169 (PMC7445397; doi:10.3762/bjoc.16.169)
Supplement: File 1 — Experimental part. [file Beilstein_J_Org_Chem-16-2026-s001.pdf]

BEILSTEIN JOURNAL OF ORGANIC CHEMISTRY

Supporting Information

for

The biomimetic synthesis of balsaminone A and ellagic acid via oxidative dimerization

Sharna-kay Daley and Nadale Downer-Riley

Beilstein J. Org. Chem. **2020,** *16,* 2026–2031. doi:10.3762/bjoc.16.169

Experimental part

License and Terms: This is a supporting information file under the terms of the Creative Commons Attribution License (http://creativecommons.org/licenses/by/4.0). Please note that the reuse, redistribution and reproduction in particular requires that the authors and source are credited.
The license is subject to the *Beilstein Journal of Organic Chemistry* terms and conditions: (https://www.beilstein-journals.org/bjoc)

Experimental

The structures of the compounds synthesized (as described below) were confirmed using ^{1}H NMR, ^{13}C NMR and infrared spectroscopy. The NMR spectra were recorded in deuterochloroform or methanol-d_4, using 200 and 500 MHz Bruker Avance instruments at The University of the West Indies, Mona, Jamaica. The resonance units are in δ (ppm) downfield of the internal standard TMS. Melting points (uncorrected) were determined on a Gallenkamp instrument. IR spectra were determined at The University of the West Indies, Mona, Jamaica, using a Tensor 37 Bruker instrument with units in wave numbers (KBr) cm^{-1}. Each reaction was monitored using thin layer chromatography (TLC) with adsorbed silica as stationary support provided by Sigma Aldrich, Madison WI, USA. Purification of the compounds synthesized was done using flash column chromatography with silica as the stationary phase.

Biomimetic Synthesis of Balsaminone A (4):

2-Methoxy-1,4-naphthoquinone (7)

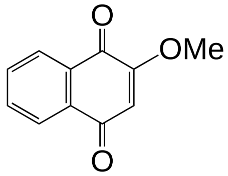

To a solution of lawsone (**6**) (166 mg, 0.955 mmol) in acetone (27 mL), was added potassium carbonate (330 mg, 2.39 mmol) followed by MeI (0.1 mL, 1.43 mmol). The mixture was stirred for 18 hours at room temperature, filtered and the solvent was removed in vacuo. The residue was dissolved in ethyl acetate (25 mL), washed with water (2 × 15 mL) and brine (2 × 15 mL), dried over magnesium sulfate, filtered and the solvent was removed in vacuo. Recrystallization of the

crude material from ethanol yielded 2-methoxy-1,4-naphthoquinone (**7**) as a yellow solid (155 mg, 86%): Mp 182 °C (lit.[i] 182 -184 °C); IR ν_{max}/cm^{-1} 837, 1006, 1669; ^{1}H NMR (200 MHz, $CDCl_3$): δ 4.07 (3H, s, OMe), 6.34 (1H, s, H-3), 7.58 (2H, m, H-6,7), 7.88 (2H, m, H-5,8); ^{13}C NMR (50 MHz, $CDCl_3$): δ 56.4, 109.8, 126.0, 126.5, 130.9, 131.9, 133.63, 134.3, 162.5, 179.9, 184.7.

General method for the oxidative dimerization using CAN, $FeCl_3$·$6H_2O$, $FeCl_3$·SiO_2:

In three portions, the oxidant (1.65 mmol) was added over 10 minutes to a rapidly stirring solution of 1,2,4-trimethoxynaphthalene (**17**) (0.5 mmol) in dry methanol (2 mL). The mixture was stirred at room temperature for 8–24 hours. After completion based on TLC, the solvent was removed in vacuo, and the organic material extracted using methylene chloride (3 × 15 mL). The organic layers were combined, dried over magnesium sulfate and the crude material purified *via* flash column chromatography (ethyl acetate-hexane 1:4).

General method for the oxidative dimerization using CAN (aq.), CrO_3, V_2O_5:

To a solution of 1,2,4-trimethoxynaphthalene (**17**) (0.5 mmol) dissolved in acetonitrile (2 mL) was added dropwise a solution of oxidant (1.65 mmol) dissolved in water (2 mL) over 30 minutes. The mixture was stirred at room temperature overnight. The acetonitrile-water mixture was reduced to 1/3 its original volume and ethyl acetate added to extract the organic material. The organic layer was filtered through neutral alumina and the solvent removed in vacuo. The crude material was purified via flash column chromatography (ethyl acetate-hexane 1:4).

General method for the oxidative dimerization using PIDA in BF_3·OEt_2, PIFA in BF_3·OEt_2 and IBX in BF_3·OEt_2:

1,2,4-Trimethoxynaphthalene (**17**) (0.5 mmol) was dissolved in dichloromethane (2 mL) at 0 °C to which the oxidant (0.75 mmol) dissolved in boron trifluoride·diethyl etherate (0.75 mmol) was added under nitrogen and the mixture stirred at room temperature for 16–36 hours, with the exception of IBX in BF_3·OEt_2 (Note 1). The reaction was quenched with saturated aqueous sodium bicarbonate (3 mL) and extracted with dichloromethane (2 × 10 mL). The organic layers were combined, dried over magnesium sulfate, and the crude material purified via flash column chromatography (ethyl acetate-hexane 1:4).

Note 1: The reaction with IBX in BF_3·OEt_2 was stirred at room temperature for 12 hours, then heated to reflux for 24 hours.

1,1′,3,3′,4,4′-Hexamethoxy-2,2′-binaphthalene (18)

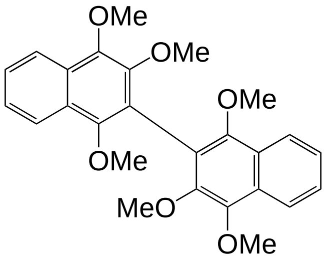

Obtained as a purple solid from the oxidative dimerization of 1,2,4-trimethoxynaphthalene (**17**)

Oxidant	Time (h)	Yield of **18** (%)
CAN	16	55
$FeCl_3$·SiO_2	16	43
$FeCl_3$·$6H_2O$	24	8

PIDA in $BF_3{\cdot}OEt_2$	24	59
PIFA in $BF_3{\cdot}OEt_2$	24	63

Method for the oxidative dimerization using $SnCl_4$

To a solution of 1,2,4-trimethoxynaphthalene (**17**) (107 mg, 0.5 mmol) in CH_2Cl_2 (1 mL) was added $SnCl_4$ (0.1 mL, 0.8 mmol) in CH_2Cl_2 (1 mL). The solution was stirred at 100 ^oC in a sealed tube for 8 hours. The reaction mixture was cooled to room temperature and saturated aqueous sodium bicarbonate (1 mL) added. The crude product was extracted with dichloromethane (2 × 10 mL), dried over magnesium sulfate and purified *via* flash column chromatography (ethyl acetate-hexane 1:4), affording biaryl **18** as a purple amorphous solid in 48% yield.

Mp 124-127 ^oC (ethyl acetate-hexane) (lit.[ii] 124 ^oC); IR ν_{max}/cm^{-1} 775, 1346, 1589; ^1H NMR (200 MHz, MeOD): δ 3.68 (6H, m, OMe), 3.77 (6H, m, OMe), 3.94 (6H, m, OMe); 7.57 (4H, m, H-6,7,6′,7′), 8.19 (4H, m, H-5,8,5′,8′); ^{13}C NMR (50 MHz, MeOD): δ 58.6, 59.7, 59.9, 104.0, 122.5, 123.6, 124.1, 126.9, 127.0, 132.1, 139.4, 149.2.

3,3′-Dimethoxy-[2,2′-binaphthalene]-1,1′,4,4′-tetrone (16)

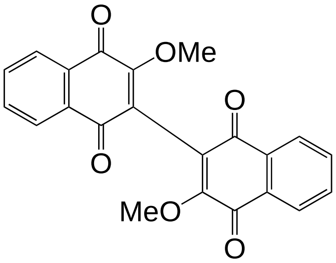

Obtained as a yellow solid from the oxidative dimerization of 1,2,4-trimethoxynaphthalene (**17**).

Oxidant	Time (h)	Yield of **16** (%)
CAN (aq.)	8	29
V_2O_5	8	34
$FeCl_3 \cdot SiO_2$	16	15
CrO_3	24	19
PIDA in $BF_3 \cdot OEt_2$	24	21
PIFA in $BF_3 \cdot OEt_2$	24	12
IBX in $BF_3 \cdot OEt_2$	36	13

Mp 195-196 °C (ethyl acetate-hexane) (lit.[iii] 194-197 °C); IR ν_{max}/cm^{-1} 1032, 1301, 1666; ^{1}H NMR (500 MHz, $CDCl_3$): δ 3.91 (6H, s, OMe), 7.36 (4H, m, H-5,8, 5',8'), 8.16 (4H, m, H-6,7,6',7'); ^{13}C NMR (125 MHz, $CDCl_3$): δ 60.9, 123.5, 126.1, 126.3, 131.2, 131.6, 133.5, 133.8, 157.6, 181.2, 182.4.

Method 2: To a solution of 2-methoxy-1,4-naphthoquinone (**7**) (320 mg, 1.69 mmol) in methanol (20 mL) was added 10% Pd/C (75 mg). The mixture was shaken under an atmosphere of hydrogen at 15 psi for 3 hours, filtered over Celite, transferred to a round bottom flask (50 mL) and the solvent removed in vacuo. The crude material was dissolved in a mixture of $CHCl_3$ (5 mL) and 30% aqueous H_2O_2 (0.1 mL), after which $SnCl_4$ (0.2 mL, 1.7 mmol) and 30% H_2O_2 (0.1 mL) were added. The mixture was stirred under nitrogen at room temperature for 10 minutes, heated at reflux for 21 hours, then cooled to room temperature and poured into water (50 mL) and 10% aqueous HCl (5 mL). The crude material was extracted with $CHCl_3$ (3 × 20 mL), and the organic layers combined and washed with water (2 × 15 mL), followed by brine (1 × 10 mL). The solution was dried over magnesium sulfate, filtered and the solvent removed in vacuo. Purification via column

chromatography using the solvent system methylene chloride-hexane (1:4), resulted in the isolation of the product **16** as a dark yellow solid (70 mg, 22%).

Method 3: To an ice-cold solution of 1,1′,3,3′,4,4′-hexamethoxy-2,2′-binaphthalene (**18**) (104 mg, 0.257 mmol) in MeCN (5 mL), was added dropwise an ice-cold solution of CAN (469 mg, 0.857 mmol) in H_2O (5 mL). The mixture was stirred for 10 minutes at 0 ^oC. The crude material was collected via vacuum and recrystallized from ethyl acetate-hexane (1:9) to afford binaphthoquinone **16** as a dark yellow solid (95 mg, 99%).

Balsaminone A (4)

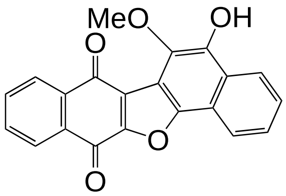

To a solution of dimethoxybinaphthoquinone **16** (103 mg, 0.275 mmol) in diethyl ether (10 mL) under nitrogen was added sodium dithionite (73 mg, 0.41 mmol) and triethylamine (0.5 mL). The mixture was stirred vigorously at room temperature under nitrogen for 24 hours, quenched with water (10 mL), and the organic material extracted using ethyl acetate (3 × 20 mL). The organic layers were combined and washed with brine (1 × 10 mL) and water (10 mL). The crude solution was dried over magnesium sulfate, filtered and the solvent removed in vacuo. Balsaminone A (**4**) was obtained as a red solid (12 mg, 13%) and the unreacted starting material recovered (64 mg, 62%), after purification *via* column chromatography using methylene chloride-hexane (1:2). Mp 273-275 ^oC (lit.[iv] 270-272 ^oC); IR ν_{max}/cm^{-1} 810, 1444, 1673; ^1H NMR (500 MHz, $CDCl_3$): δ 4.12, (3H, s, OMe), 7.69-7.30, (2H, m, H-2,3), 7.88-7.90 (2H, m, H-1,4), 8.35-8.41 (2H, m, H-9,10), 8.55-8.61 (2H, m, H-8,11); ^{13}C NMR (125 MHz, $CDCl_3$): δ 62.9, 113.0, 118.1, 120.6, 122.4, 124.3, 124.9, 126.7, 127.1, 127.6, 127.9, 131.6, 133.0, 133.6, 134.6, 143.4, 148.1, 153.0, 174.7, 180.2.

Biomimetic synthesis of ellagic acid (4):

Methyl gallate (15)

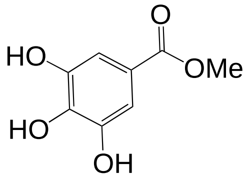

To a solution of gallic acid (**10**) (3.18 g, 18 mmol) in dry methanol (30 mL) was added sulfuric acid (0.3 mL). The mixture was heated at reflux for 6 hours, cooled to room temperature and the solvent removed *in vacuo* to afford the crude natural product (3.3 g, quant.). Recrystallization from water afforded methyl gallate (**15**) as white crystals (1.09 g, 33%). Mp 204-207 ^{o}C (lit.[v] 201-204 ^{o}C); ^{1}H NMR (500 MHz, MeOD): δ 3.77 (3H, s, OMe), 6.89 (2H, s), 7.02 (3H, br, OH); ^{13}C NMR (125 MHz, MeOD): δ 51.9, 109.8, 121.8, 138.8, 146.0, 167.2.

<u>Dimethyl 4,4′,5,5′,6,6′-hexahydroxy-[1,1′-biphenyl]-2,2′-dicarboxylate (19)</u>

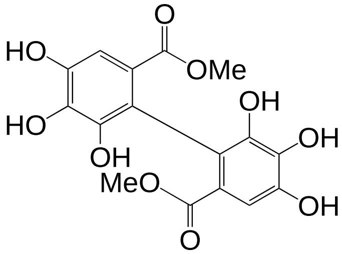

Obtained as a cream solid from the oxidative dimerization of methyl gallate (**15**). See general method for the oxidative dimerization using PIDA, CAN, $FeCl_3·SiO_2$ and $FeCl_3·6H_2O$. Purification of the crude product was done via flash column chromatography with methanol-dichloromethane (0.5:9) as eluent.

Oxidant	Time (h)	Yield (%)
PIDA in $BF_3 \cdot OEt_2$	16	80
CAN	16	54
$FeCl_3 \cdot SiO_2$	24	49
$FeCl_3 \cdot 6H_2O$	24	33

Mp 240-242 °C (methanol) (lit.[v] 240-245 °C); IR ν_{max}/cm^{-1} 872, 1078, 1669, 3089, 3550; ^{1}H NMR (500 MHz, MeOD): δ 3.44 (3H, s, OMe), 6.99 (2H, s), 7.54 (6H, br, OH); ^{13}C NMR (125 MHz, MeOD): δ 51.3, 110.7, 118.7, 112.7, 137.5, 144.2, 144.7. 167.4.

<u>Ellagic acid (5)</u>

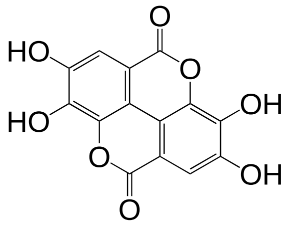

Method 1: To a solution of methyl gallate (**15**) (128 mg, 0.69 mmol) dissolved in methylene chloride (10 mL), was added PIFA (455 mg, 1.06 mmol) dissolved in boron trifluoride-diethyl etherate (1.5 mL). The reaction mixture was stirred at ambient temperature under nitrogen for 24 hours. Water (10 mL) was added to quench the reaction mixture. Extraction using *n*-butanol (4 × 10 mL), followed by purification *via* column chromatography (acetone-hexane 1:4), afforded the natural product **5** as a cream solid (87 mg, 83%). Mp 350-351 °C (methanol-water) (lit.[ii] 350 °C); IR ν_{max}/cm^{-1}1055, 1725, 2850, 3022, 3569; ^{1}H NMR (500 MHz, MeOD): δ 7.43 (2H, s); ^{13}C NMR (125 MHz, MeOD): δ 107.6, 110.2, 112.3, 136.4, 139.6, 148.0, 159.1.

Method 2: A solution of dicarboxylate **19** (72 mg, 0.19 mmol) was heated at reflux in methanol/water (1:1, 3 mL) for 24 hours, then cooled to room temperature. The crude material was extracted with *n*-butanol (3 × 10 mL) and the solvent evaporated in vacuo. Recrystallization of the crude material with DMSO-chloroform (1:1) afforded the natural product **5** as a cream solid (58.7 mg, 100%).

---

[i]. Wang, C.; Li, W.; Wu, D.; Wang, J.; Wu, C.; Liao, J.; Lin, C. *Evid.-Based Compl. Alt. Med.* **2011**, Art. ID 704721, pp 8.
[ii]Laatsch, H. *Z. Naturforsch*. **1990**, *45B*, 393-400.
[iii] Qi, C.; Wang, W.; Reichl, K. D.; McNeely, J.; Porco, Jr., J. A. *Angew. Chem., Int. Ed.* **2018**, *57*, 2101-2104.
[iv] Padwal, W.; Lewis, W.; Moody, C. J. *J. Org. Chem.* **2011**, *76*, 8082-8087.
[v] Alam, A.; Takaguchi, Y.; Tsuboi, A. *J. Fac. Environ. Sci. Technol., Okayama Univ.* **2005**, *10*, l11-117.
